# McGill University, Comparative Psychology Society

**Published:** 1896-04

**Authors:** 


### PROCEEDINGS FOR THE YEAR 1894-95 OF THE SOCIETY FOR THE STUDY OF COMPARATIVE PSYCHOLOGY IN CONNECTION WITH THE FACULTY OF COMPARATIVE MEDICINE AND VETERINARY SCIENCE OF McGILL UNIVERSITY, MONTREAL.

(Continued from page 78.)

After the reading of the minutes of the previous meeting, Dr. Mills was tendered a vote of thanks by the Society for his recent contribution to the Library.

Mr. W. K. Inglis then read a paper on "Expression of Emotion in the Lower Animals," which evidenced on the part of the writer much original thought and investigation. Darwin has described three principles which probably account for most of the expressions and gestures of the lower animals, and these were considered in order. The first principle is that of serviceable associated habits, and it was shown that when a certain state of mind is induced there is a strong tendency, through the force of habit and association, to a recurrence of that state and the performance of related movements, though they may be of no use to the animal, and thus the most complex movements can in time be performed without any effort or even consciousness. Physiology explains how this occurs, in that the conducting power of various paths is increased according to the frequency of the impulses which pass along them. Thus certain paths become associated with certain nervous impulses, and this association applies to the nerves of motion, sensation, and those connected with thinking. Some psychical change probably occurs, for the tendency to acquired habit is inherited. If one of a series of associated habitual movements be performed, the other will naturally follow. Some of these may be useless, but having originally been performed for some definite purpose under different environment they still remain. For instance, dogs frequently turn around several times and even scratch the ground before lying down, which useless habit is, no doubt, a remnant of one that was the opposite in their parents in a wild state, having for its object the making of a comfortable resting-place in the long grass. Certain mental conditions lead to certain actions, but when a directly opposite condition is induced there is a strong and involuntary tendency to the performance of actions of an entirely opposite nature. This is the second principle—that of antithesis. The development of movements under this principle are, according to Darwin, dependent upon consciousness. Certain movements have required certain sets of muscles for their performance, and movements of an opposite nature have brought other sets of muscles into play. "So strongly are our intentions and movements associated together that, if we eagerly wish for an object to move in any direction, we can hardly avoid moving our bodies in the same direction, although we may be perfectly aware that this can have no influence." The third principle refers to actions of nervous origin, but entirely independent of the will, and, to a certain extent, of habit. The brain, under certain conditions, generates and transmits nervous impulses in certain directions in excess, depending on the connection of the nerve cells with the fibres, and, as far as the muscular system is concerned, on the nature of the movements which have been habitually practised. As examples of this principle we have trembling of the muscles, chattering of the teeth, and the regulation of the capacity of the arteries by the vaso-motor system might also be included. The means of expression in the different groups of animals was next considered. Cattle and horses seldom emit sounds when suffering pain unless it be excessive, but cows invariably bellow after their offspring is removed, and a horse will call to his mate when separated or on his return. The variety of emotions which the dog can express by sounds has been commented on by most writers on animal intelligence, and is apparent to anyone who will devote a little attention to the matter. In the pig the principle of antithesis is evidently concerned in the difference between his contented grunt after his appetite has been appeased and the terrified squeal when roughly handled. But in the lower animals the expression of emotions is accomplished for the most part by other methods than sounds. The erection of the dermal appendanges, as the hair and feathers, may be characteristic of fear or anger, while the action of the tail, of the dog in particular, as an evidence of certain mental states, needs scarcely to be mentioned. The ears in many animals, especially the horse, are indicative of emotion. Horses in a wild state, when fighting, draw the ears back to prevent their being seized by their antagonist, and the habit still obtains in the domesticated animal. When pleased or interested they assume the reverse position. Cattle and sheep do not display as much emotion as horses and dogs. The paper was brought to a close by a brief reference to the importance of the subject of expressions from a comparative point of view.

A profitable discussion followed. Mr. Cutting asked, "Why do dogs roll in carrion?" In reply the President stated that some dogs will continue it after being punished, but all dogs are not equally prone to the habit. It is probably inherited from some of the early progenitors, who, having at some time killed an animal, return to the spot and roll over the carcass to associate the sensation. Mr. Baldwin suggested that the habit may have had its origin in the fact that having killed an animal the dog would roll to obtain the scent, and thus, perhaps, attract others. Mr. Zink thought it quite possible that this odor, though unpleasant to us, might be the reverse in the dog. Mr. Cleaves concurred in this opinion.

The President referred to some interesting facts concerning the psychic development of a kitten which he had had under observation since its birth.

Mr. Zink then read an article on "Animal Psychology," written by an American clergyman, and which recently appeared in the *Dog Fancier;* after which the meeting adjourned.

After the reading of the minutes of the previous meeting the President announced an addition to the Library of several valuable works on animal psychology and related subjects.

Mr. W. V. Jones read a paper on the "Evolution of Mind in the Lower Animals," in which he first directed attention to possible erroneous deductions regarding the mental processes of animals. Until recently, even among those who have adopted the theory of evolution as regards physical development, the idea has prevailed that mind is essentially a human attribute, but there is at present a growing tendency, born of scientific investigation, to apply the same natural law to psychic development. The growth of mind has been augmented and accelerated by the aid of language. The criterion of mind is consciousness as evidenced by the power of choice, that is, the performance of actions differing from those usually resulting from the same stimuli, but we also see in the lower animals and plants, and even in inanimate things, actions which almost appear to be the result of choice. Is it possible, then, to evolve anything of a psychic nature by the action of force upon matter? Are the atoms of matter inert things having nothing but the property of extension? The law of habit holds sway over the inanimate world also. Matter once acted upon by an external force reacts against a counter-force, and it is reasonable to suppose that when forming part of a tissue the atoms do not comport themselves differently. The potentiality of matter increases by virtue of its experience, and higher dominant centres become evolved, and these become subordinated to others, and we may even imagine a complete organism like the animal body having for its dominant centre the nervous system. The question arises: At what stage does consciousness make its appearance? We know that it involves a variety of stimuli, as one unvarying stimulus exhausts reactionary power. An entity must, therefore, have had sufficient experience to enable it to react against a variety of stimuli, and having arrived at this stage of differentiation it has two or more subordinate centres which respond to different stimuli, and these centres are under the influence of the dominant centre which possesses all the powers of the constituent parts, and, in addition, other powers which it has acquired by virtue of its greater experience. It is then just in this difference of power between the dominant part and the entity itself, in that it is capable, under certain stimuli, of influencing the entity in a way not in accord with its previous experience, causing rearrangement for the purpose of responding to a new stimulus, that we understand the first dawning of consciousness, and the human mind is but the topmost inflorescence of one mighty growth, whose roots, stems, and many branches are sunk in the abyss of planetary time.

(To be continued.)

